# Editorial: Insights in red blood cell physiology: 2023

**DOI:** 10.3389/fphys.2024.1437573

**Published:** 2024-06-05

**Authors:** Anna Bogdanova, Lars Kaestner

**Affiliations:** ^1^ Red Blood Cell Group, Institute of Veterinary Physiology, University of Zurich, Zurich, Switzerland; ^2^ Dynamics of Fluids, Experimental Physics, Saarland University, Saarbrücken, Germany; ^3^ Theoretical Medicine and Biosciences, Medical Faculty, Saarland University, Homburg, Saar, Germany

**Keywords:** sickle cell disease, drug transport, asplenia, transfusion, extracellular vesicle release, GP.Mur, COVID-19, hemorheology

The contributions of the Research Topic “Insights in Red Blood Cell Physiology: 2023” brought to our attention most relevant areas of red blood cell (RBC) research. They include the RBCs as targets and the active players defining the severity of disease manifestation in patients with SARS-CoV-2 (Lechuga et al.; Rogers et al.) and sickle cell disease (Goksel et al.), the possibilities to use RBCs as drug carriers (Biagiotti et al.), the role of RBC membrane dynamics in progression of storage lesions of RBCs (Ghodsi et al.) and in asplenic patients (Dumas et al.), as well as various aspects of optimization of oxygen delivery (Hsu; Wise et al.).

Since the first days of SARS-CoV-2 pandemics, RBC oxidation (Papadopoulos et al., 2022; Bellanti et al., 2023), anemia (Chen et al., 2021) or secondary polycythemia (Yavorkovsky et al., 2023), and increased risk of thrombosis (Vrečko et al., 2022; Bellanti et al., 2023; Farooqui et al., 2023) were reported as confounding factors associated with compromised respiratory function contributing to the severe impairment of oxygen delivery. Also changes in RBC shape and deformability were previously reported for COVID-19 patients (Kubánková et al., 2021; Recktenwald et al., 2022). In their paper Rogers et al. use Lorrca Maxsis to assess the changes in rheological parameters and aggregation index caused by SARS-CoV-2 infection in patients with A and O blood groups and investigated the impact of disease on oxygen transport by hemoglobin. The obtained results reveal the alterations in RBC rheology and oxygen transport capacity in patients with COVID-19 questioning the “protective role” of the blood group O that was claimed by other studies to be associated with milder disease phenotype. A review article by Lechuga et al. summarizes the recent updates on the alterations in RBC caused by “long COVID-19” when symptoms prevail 12 weeks after the disease onset. It is complementary to the review of Papadopulos et al. which focuses on the changes in RBC properties during the acute COVID-19 phase (Papadopoulos et al., 2022). The impact of the interaction of erythroid precursor cells and the circulating cells with the virus itself and the proinflammatory cytokines on RBC indices, deformability, and morphology as well as on the iron metabolism is discussed. Transfusion is a common measure to correct for anemia, also for COVID patients. However, both unstable RBC membranes and stored RBCs release vesicles that may induce a hypercoagulation state (Leal et al., 2018). In their study, Ghodsi et al. show that RBCs of some donors are more prone to vesiculation during storage than those of others. The authors revealed that the two groups of donors differ in the abundance of cholesterol content of plasma membranes suggesting that cholesterol-rich domains are focal points for the onset of vesiculation. Another type of vesiculation is associated with the release of organelles from reticulocytes during their maturation (Dumas et al.). In this case, theese “vacuoles” are formed that encapsulate the organelles inside the cell and then released from the cells in the process requiring passages through the spleen.

Vesiculation is a challenge for those who try to use RBCs as delivery systems for cytotoxic drugs (Wang et al., 2023). A review dedicated to the recent developments in using RBCs as carriers for drug transport is presented by Biagiotti et al. The optimization of delivery and the possible applications of this delivery system are discussed including modulators of oxygen delivery and immunosuppressive drugs, chemotherapeutics, anti-diabetic and psychoactive drugs.

RBCs are a natural carrier of the compounds regulating vascular tone. The group of Hsu explores the role of anion exchanger-1 in the transport of nitrite into RBCs, where it is reduced to NO by deoxyhemoglobin and released from the cells when they face hypoxic microenvironment causing vasodilatation. Wise et al. discuss plasticity in adjusting oxygen delivery by RBCs from the lungs to hypoxic peripheral tissues by combining vaso-regualtory action with precise control over hemoglobin oxygen affinity. Modulators of hemoglobin’s oxygen affinity decreasing the concentration of 2,3-bisphosphoglycerate are currently trialed as symptomatic therapy for patients with sickle cell disease (Parekh et al., 2024). In contrast, from a high-altitude study, it was suggested that hypoxia modulates 2,3-bisphosphoglycerate levels in human RBCs (D’Alessandro et al., 2024). Goksel et al. suggest that inhibitors of phosphodiesterase PDE1 may further contribute to the optimization of oxygen delivery in sickle cell disease patients by making RBC more deformable when exposed to shear stress.

Having a compilation of contributions dealing with basic physiological principles (Wise et al., Hsu), relating to diseases with direct and indirect involvement of RBCs (Goksel et al.; Dumas et al.; Rogers et al.; Lechuga et al.), concerning transfusion medicine-related topics (Ghodsi et al.), and reviewing therapeutic approaches (Biagiotti et al.), we are convinced the series of “Insights in Red Blood Cell Physiology” appearing since 2021 (Kaestner and Bogdanova, 2022) is of utmost relevance and will continue in future.
